# Preparing for successful protein crystallization experiments

**DOI:** 10.1107/S2053230X25004650

**Published:** 2025-06-02

**Authors:** Gabrielle R. Budziszewski, Vivian Stojanoff, Sarah E. J. Bowman

**Affiliations:** a University at Buffalo Hauptman Woodward Institute, Buffalo, NY14203, USA; b Department of Biochemistry, Jacobs School of Medicine and Biomedical Science, University at Buffalo, Buffalo, NY14203, USA; c National Synchrotron Light Source II Brookhaven National Laboratory Upton NY11973 USA; University of Pennsylvania, USA

**Keywords:** crystallization, X-ray crystallography, structural biology, X-ray free-electron lasers, electron diffraction

## Abstract

Crystal-based structural methods, including X-ray crystallography, are frequently utilized for the determination of high-resolution structures of biomolecules. All crystal-based diffraction methods first require the preparation of biomolecular crystals, and careful sample preparation for crystallization experiments can increase the frequency of success. In this article, strategies to optimize factors that can impact crystallization are presented, from which buffers and reducing agents are most favorable to which crystallization techniques could be used.

## Abstract 2

All crystal-based structural biology methods, including X-ray crystallography, serial synchrotron and serial femtosecond crystallography, and electron diffraction, require the preparation of biomolecular crystals. This article covers strategies for careful sample preparation in crystallization experiments to increase the chance of success.

## 1. Introduction: what is crystallization?

Crystallization is the first step in performing any crystal-based diffraction experiment. Close to 85% of all biomolecular structural models deposited in the Protein Data Bank (PDB) are from crystal-based experiments (Budziszewski *et al.*, 2023 ▸), highlighting the importance of crystallization as a method. At its core, biomolecular crystallization is a process in which crystals grow from a thermodynamically metastable or supersaturated solution via a phase-separation process. Crystallization of biomolecules requires a balance between stabilizing and solubilizing the sample coupled with driving toward an ordered aggregate, resulting in a lattice held together by a periodic network of sparse and weak intermolecular inter­actions. For biomolecules, crystallization often proceeds through extensive experimentation, but there are guiding principles that can be employed to increase the chance of success. There is an extensive history of literature that describes methods to crystallize biomolecules (Luft *et al.*, 2001 ▸; Dale *et al.*, 2003 ▸; Bergfors, 2009 ▸; McPherson & Gavira, 2014 ▸; Rosa *et al.*, 2020 ▸; Lynch *et al.*, 2023 ▸). Here, we build upon that literature to discuss specific features of sample preparation that should be considered prior to biomolecular crystallization experiments, as described during the workshop entitled SAMPREP (Sample Attributes for Multiple techniques and Principal Requirements for Experiments in Pan-structural biology) presented during the 73rd Annual American Crystallographic Association Meeting in July 2023.

## 2. Biochemical considerations

There are a wide variety of biochemical parameters to consider when preparing a sample for crystallization experiments. Firstly, a high level of purity (typically >95%) is needed for biomolecules to crystallize. Methods to investigate the purity of samples used in structural studies have been discussed (Liu *et al.*, 2020 ▸). Sources of impurities and heterogeneity that may impact crystallization include oligomerization, isoforms, flexible regions, disordered regions, misfolded populations, partial proteolysis, cysteine oxidation and deamidation of Asn and Gln residues to Asp and Glu residues. Additional biochemical considerations include the presence or absence of glycosylation or post-translational modifications. If crystals do form in the presence of impurities, the result is often poor diffraction due to a disordered crystal lattice.

Secondly, the biomolecular sample needs to be very stable for crystallization, as crystals can take an extended time (days to months) to nucleate. Components to consider to maintain sample stability include buffers, salts, glycerol and substrates for soluble proteins, in addition to detergents, micelles or nanodiscs for membrane proteins. Ideally, buffer components should be kept below ∼25 m*M* concentration and salt components (*i.e.* sodium chloride) should be kept below 200 m*M* concentration. Phosphate buffers should be avoided, as they easily form insoluble salts. Some samples will require addition of substrate, ligand, coordinating metal or reductant to the sample buffer to keep the biomolecule stable. When using chemical reductants during crystallization, reductant lifetime should be considered in the context of the timescale for crystal growth (Table 1 ▸). Methods such as differential scanning fluorimetry and circular dichroism can be used to assess the stability of the sample as a function of buffer component to identify the most suitable buffer, salt and pH, as well as to investigate the impact of temperature and the presence of ligands or stabilizing chemicals on stability. The ideal pH is one at which the sample is stable, as surface charges can affect crystal packing. Note that the crystallization cocktail (see below) is also pH dependent, and pH is a common cocktail parameter to vary during crystallization optimization.

Thirdly, a highly soluble, homogeneous sample is usually required for optimal crystallization experiments. A number of approaches are appropriate to assess sample homogeneity and solubility, including dynamic light scattering (DLS), size-exclusion chromatography (SEC), size-exclusion chromatography coupled with multi-angle light scattering (SEC-MALS) and mass photometry. An ideal sample for crystallization will be monodisperse and not prone to aggregation. Glycerol is often needed for sample solubilization. Practically speaking, for crystallization trials glycerol should be kept to below 5%(*v*/*v*) in the final crystallization drop. The best sample buffer to use in crystallization trials is the simplest formulation that will maintain sample stability, solubility and activity. Once the best conditions have been determined for the sample, crystallization experiments can proceed.

All of the considerations above can be, in part, addressed by carefully considering construct design prior to, or iteratively with, crystallization screening. Once the structural objective has been honed, constructs can be analyzed for stability and crystallization propensity (Slabinski *et al.*, 2007 ▸). Flexible regions are generally unfavorable to crystallization, as they induce conformational heterogeneity. *AlphaFold*3 is an excellent resource to guide construct design and eliminate floppy regions that may interfere with crystallization (Abramson *et al.*, 2024 ▸). Affinity tags can improve the solubility properties of some proteins and act as crystallization chaperones (Smyth *et al.*, 2003 ▸; Tamura *et al.*, 2019 ▸; Nawarathnage *et al.*, 2023 ▸) and may be worthwhile pursuing in challenging cases. Biomolecules that continue to prove recalcitrant to crystallization may also be resurfaced to improve crystal contacts (Derewenda & Vekilov, 2006 ▸; Liu *et al.*, 2007 ▸; Banayan *et al.*, 2024 ▸), but care should be taken to validate that mutations do not broadly disrupt structure or function in a way that can invalidate conclusions from structural data.

## 3. Physical considerations

**Figure 1:**
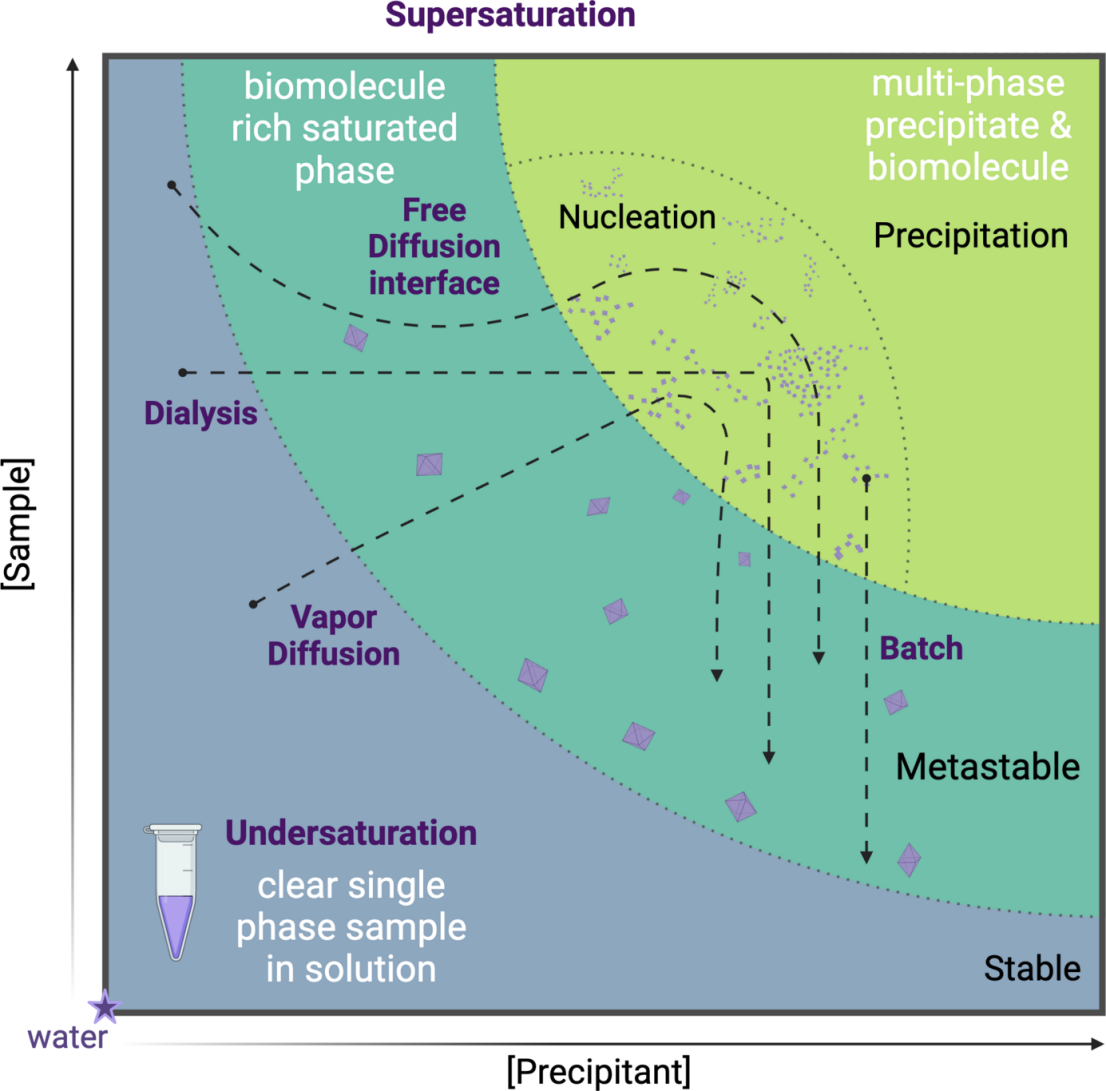
A phase diagram for biomolecular crystallization, with the sample concentration on the *y* axis and crystallization precipitant (cocktail) on the *x* axis. Different methods of crystallization can be utilized to assist biomolecular crystal nucleation, followed by stabilization in the metastable zone suitable for structural studies.

Crystallization typically occurs in the presence of a cocktail of chemical components that promote crystal formation (often referred to as the crystallization cocktail or mother liquor). These chemical mixtures are designed to modulate the solubility of biomolecules. A crystallization cocktail that promotes productive crystal formation will cause the sample to traverse the phase diagram from the undersaturated phase into the nucleation and metastable phases (Fig. 1 ▸). Crystallization conditions are generally composed of some combination of buffers to mediate pH, salts, polymers and additives. Crystallization of biomolecules remains primarily determined empirically, so many trials of crystallization conditions may be required to find a suitable condition to induce crystal growth. As a starting point, crystallization conditions for homologous proteins can be extracted from the PDB. While efforts have been made to mine the data in the PDB to generate predictive algorithms for protein crystallization (Lynch *et al.*, 2020 ▸; Abrahams & Newman, 2019 ▸), often an empirical approach proves to be necessary. Furthermore, crystallization components can impact features of the crystal lattice, including crystalline order, solvent content and space group, which are key considerations for the diffraction experiment and downstream structural interpretation.

One mechanism by which crystallization components influence biomolecule solubility is through the salting-out phenomenon (McPherson, 2001 ▸; Finet *et al.*, 2003 ▸). Up to a certain salt concentration, salt molecules will enhance biomolecule stability by generating electrostatic contacts with the protein surface. However, when salt concentrations rise past a certain threshold, salts begin to compete with the biomolecule for access to water molecules, forcing biomolecules to favor the weaker intermolecular interactions that lead to lattice formation and crystal packing. The concentration of salt at which salting-out occurs is biomolecule- and salt-dependent. Ammonium sulfate is one commonly used salt for protein crystallization (Dumetz *et al.*, 2007 ▸), so much so that ammonium sulfate screens are offered by major crystallization screen suppliers. Salts are common crystallization condition components, and participate not only in reducing biomolecule solubility, but also in binding to biomolecules as active ligands, particularly in the case of metal salts, as well as mediating intermolecular interactions between biomolecules in the crystal lattice.

Polymers are another commonly used crystallization component, and serve several functions that can promote the crystallization of biomolecules (Finet *et al.*, 2003 ▸; Anderson *et al.*, 2006 ▸; Lynch *et al.*, 2020 ▸). One function is to screen salt-mediated aggregation at high salt concentrations that may lead to unproductive precipitation of biomolecules (Ray & Puvathingal, 1986 ▸). Polymers, such as high-molecular-weight polyethylene glycols (PEGs), are also thought to induce macromolecular crowding (Lynch *et al.*, 2020 ▸; Rastogi & Chowdhury, 2021 ▸; Liebau *et al.*, 2024 ▸), increasing the likelihood of biomolecules encountering one another in solution in a manner befitting an ordered lattice. Viscosity could play a role in reducing the entropic motion of biomolecules, perhaps lowering the barrier to crystal lattice formation. Polymers and some salts at significant concentrations also serve as cryoprotectant molecules, promoting the formation of vitreous ice during cryocooling crystals.

Buffers to control the pH of the crystallization condition are often desirable, as biomolecules frequently prefer to crystallize within 1–2 pH units of their pI (Kantardjieff & Rupp, 2004 ▸). The pH of the solution impacts the ionization state of ionizable amino acids, which can promote or antagonize intermolecular interactions. Additives that promote biomolecule stability are generally beneficial, and may influence the ordering of floppy regions of biomolecules or mediate intermolecular interactions required for lattice formation.

Additives are often key to inducing biomolecular crystallization. The most common additive is 2-methyl-2,4-pentanediol (MPD), which binds to hydrophobic protein regions and affects the overall hydration shell of the biomolecule (Anand *et al.*, 2002 ▸). Useful additives may also include cofactors, substrate molecules, nonhydrolyzable substrates, small molecules, partner proteins and Fab fragments that bind the target biomolecule (Hoeppner *et al.*, 2013 ▸; Griffin & Lawson, 2011 ▸; McPherson & Cudney, 2006 ▸; Lieberman *et al.*, 2011 ▸).

Another key consideration for biomolecular crystallization is the concentration of the biomolecule in solution when conducting crystallization screening. If we consult the phase diagram (Fig. 1 ▸), there is a theoretical region where despite modulating the precipitant concentration, there is no change in phase because the sample is not adequately concentrated. Similarly, biomolecules can be overly concentrated and yield only precipitation during crystallization screening efforts rather than productive nucleation and crystal growth. Therefore, biomolecule concentration should be carefully considered before conducting large-scale crystallization screening.

Biomolecules for crystallization should be highly pure (>95%) and homogenous in their stabilizing solution. Care must be taken not to concentrate the sample beyond the limits of solubility, at which soluble and insoluble aggregates can form, interfering with the crystallization process. Useful methods for assessing the homogeneity of a protein solution include native gel electrophoresis, DLS and SEC-MALS.

To assess whether the sample concentration is appropriate, a pre-crystallization test based on the sparse-matrix crystallization approach is useful (Jancarik & Kim, 1991 ▸). The results of a hanging-drop crystallization experiment with four crystallization cocktails serve as a guide to achieving a productive concentration of biomolecule for crystallization. In rare cases experienced by the authors, pre-crystallization testing leads directly to the growth of well diffracting protein crystals!

Finding appropriate crystallization conditions remains the primary bottleneck in crystal-based structural determination methods, and therefore the likelihood of success is increased as more conditions are tested (Lynch *et al.*, 2023 ▸). The chemical space of crystallization cocktails is vast, and approaches to screen this space most effectively have been developed. These approaches include the incomplete factorial (Carter & Carter, 1979 ▸), where key drivers of crystallization are identified by limited combinations of multiple variables, and the sparse-matrix approach (Jancarik & Kim, 1991 ▸), which includes conditions which have previously been successful in crystallizing biomolecules. Many commercial screens now exist for both general biomolecular crystallization and more specialized applications, including membrane proteins, nucleic acids and nucleic acid complexes, and protein–ligand complexes.

**Figure 2:**
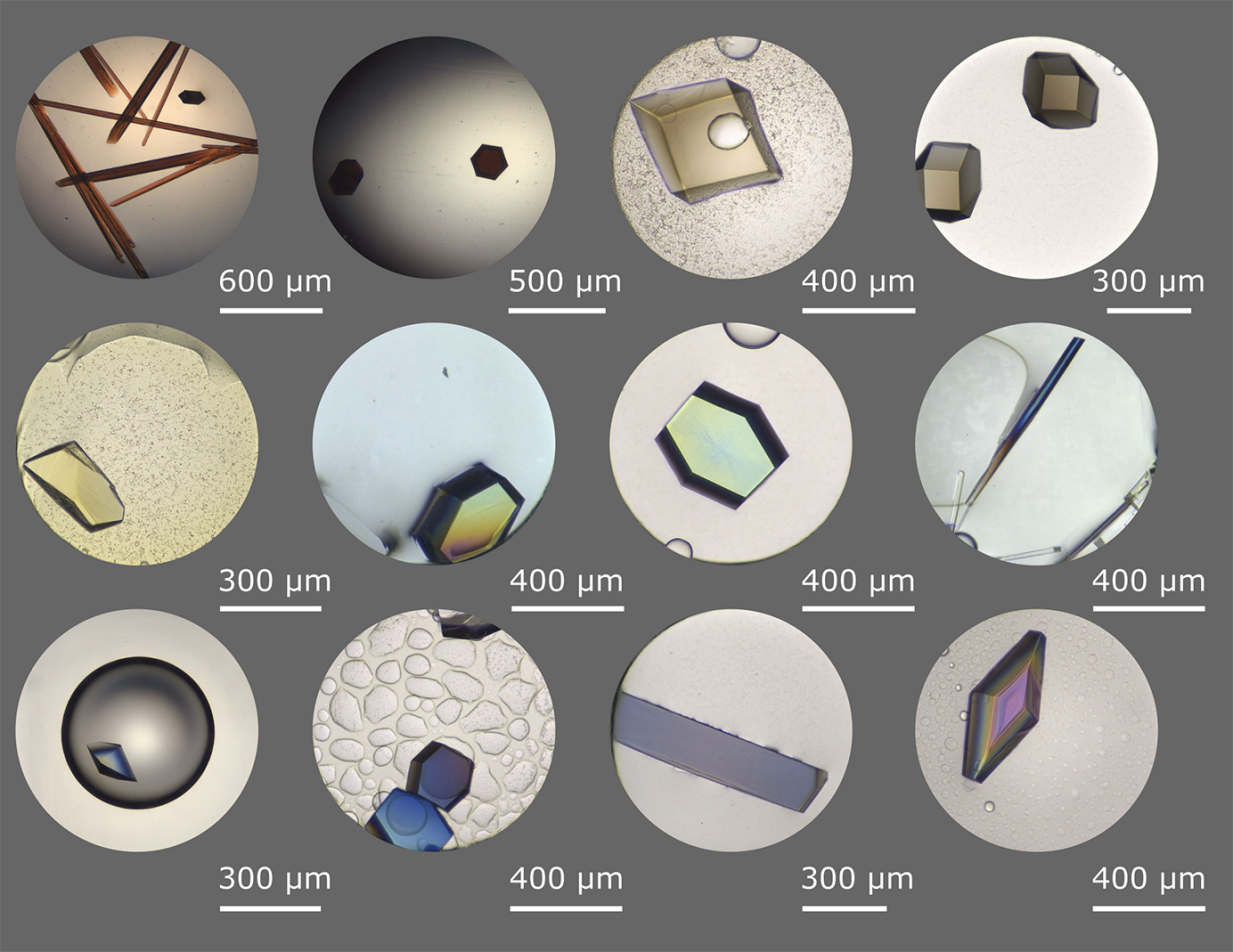
A rainbow of brightfield images of several successful crystallization ‘hits’ as examples (a scale bar is shown to the lower right of each image).

**Figure 3:**
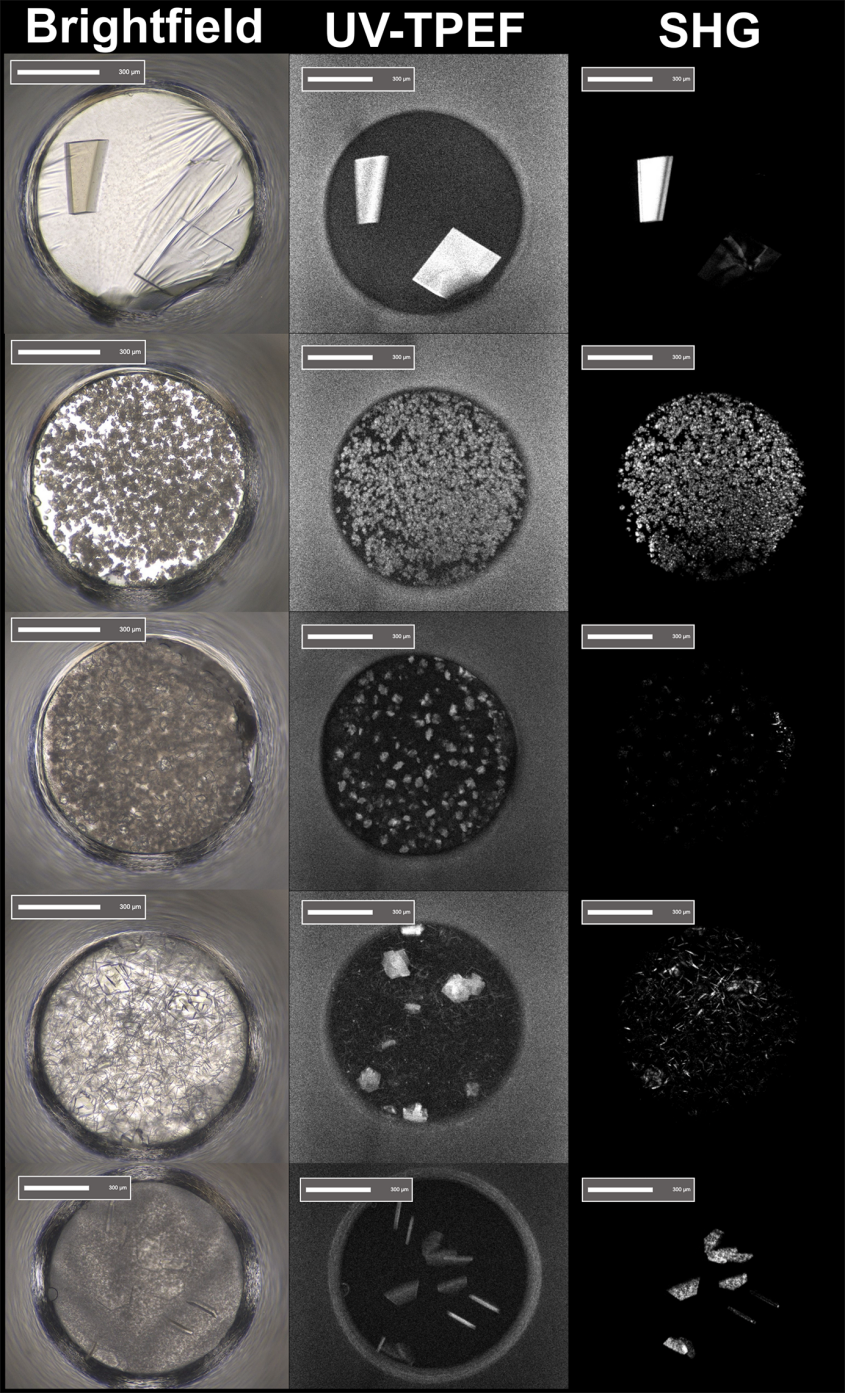
Example of five crystallization wells shown with brightfield (left), UV-TPEF (middle) and second-harmonic generation (SHG) SONICC images of successful crystallization screening using different types of imaging modalities. Even when protein crystals are obscured by precipitate, UV-TPEF and SHG images can assist with the identification of potentially promising crystal hits. These imaging methods also assist with the identification of microcrystals and nanocrystals.

Interpretation of crystallization results from screening can yield more than just positive crystallization results (‘hits’) and different crystal-based methods have different optimal crystal sizes and shapes. Most would consider a well formed single macrocrystal an excellent result of the screening process (Fig. 2 ▸). However, outcomes such as precipitation, phase separation, spherulite formation, needles, plates and microcrystals can be analyzed to gain knowledge about the phase behavior of the sample during crystallization experiments, as all represent a point on the phase diagram. Several imaging methods, including the use of UV–Vis, UV two-photon excited fluorescence (UV-TPEF) and second-harmonic generation techniques (second-order nonlinear imaging of chiral crystals; SONICC), are available to assist in the identification of protein crystals (Fig. 3 ▸; Haupert & Simpson, 2011 ▸; Lynch *et al.*, 2023 ▸). It should be noted that some macromolecules, including nucleic acids and metalloproteins, can sometimes quench the UV signal, which can lead to the dismissal of macromolecular crystals as salt if care is not taken.

Most crystallization hits require some optimization to achieve adequate crystal size, morphology and diffraction properties. Generally, optimization is conducted by iterative grid screening, wherein two chemical variables are chosen to vary above and below the hit condition concentration such that the local chemical space is well defined. Some of these variables include precipitant concentration, pH, salt concentration and additives. Additionally, the crystallization format may be altered, which influences the path through the phase diagram by which crystallization is achieved (Fig. 1 ▸). It can also be helpful to use multiple drop volume ratios of macromolecule:cocktail when optimizing conditions. Often when the format or drop size is altered conditions need to be optimized to accommodate the change. Finally, altering the temperature at which the experiment is incubated can influence both protein solubility and slow crystallization kinetics, as lowering the incubation temperature can sometimes resolve problems such as crystallization defects caused by rapid growth at higher temperatures. The most commonly used temperatures for protein crystallization are 277, 298 and 293 K (Lynch *et al.*, 2020 ▸).

Importantly, crystal quality is not always directly correlated with appearance in the drop. Many crystallographers have experienced the heartbreak of a beautiful crystal that does not diffract or the thrill of an ugly duckling that yields a high-quality diffraction data set. The results of the diffraction experiment provide information to guide the experimenter in further optimization. Some crystal pathologies can be resolved by altering the crystallization conditions or incubation temperature, but occasionally a condition results in a dead end where no further optimization can improve the crystal quality or diffraction properties. In these cases, it is appropriate to move on from these conditions or even reconsider the original biomolecular construct. Construct factors such as adding or removing affinity tags (Kuge *et al.*, 1997 ▸; Saul *et al.*, 1998 ▸; Chun *et al.*, 2012 ▸; Zou *et al.*, 2012 ▸), removing flexible regions, engineering short linkers between complex components (Aÿ *et al.*, 1998 ▸) or resurfacing the biomolecule (Derewenda, 2004 ▸; Banayan *et al.*, 2024 ▸) can be considered at the discretion of the experimenter.

## 4. Crystallization methods

**Figure 4:**
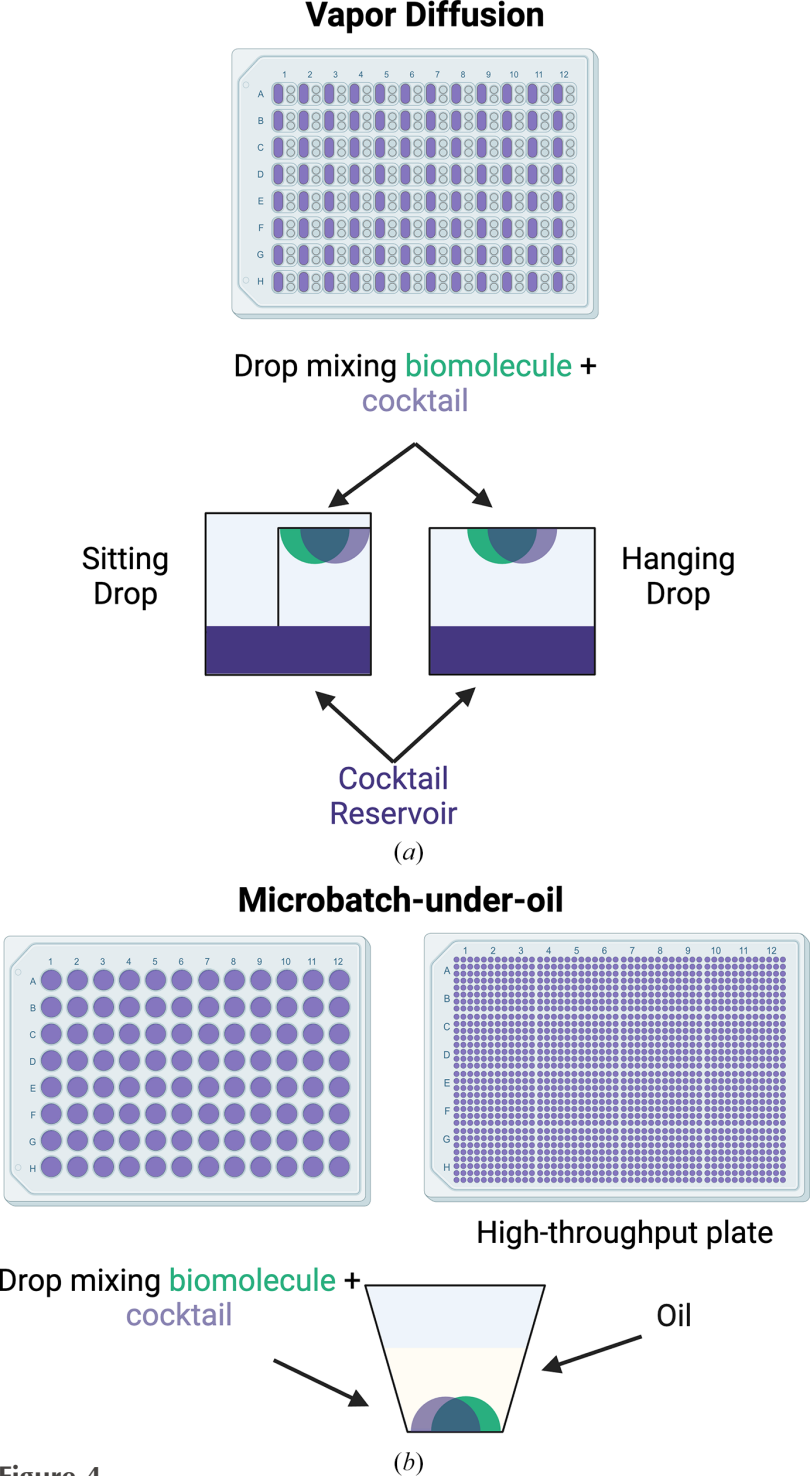
Different methods of crystallization experiment setup include (*a*) vapor diffusion and (*b*) microbatch-under-oil.

Vapor diffusion is one of the primary methods used in macromolecular crystallization experiments. In vapor diffusion, the macromolecule is combined with the crystallization cocktail and then sealed in a well with a reservoir of the cocktail (Fig. 4 ▸*a*). Vapor diffusion can be set up in a sitting-drop format or a hanging-drop format; in both cases the drops slowly dehydrate as water vapor diffuses from the drop to the reservoir (Benvenuti & Mangani, 2007 ▸). Microbatch-under-oil, another technique that is commonly used, depends on combining the macromolecule and cocktail in a drop under paraffin or mineral oil (Fig. 4 ▸*b*; Chayen *et al.*, 1992 ▸). Various plate types are available for setting up drops either manually or using robotics. Robotic liquid-handling instrumentation can assist with drop setup with very small volumes, from 25 to 200 nl, making efficient use of precious and hard-to-prepare bio­molecular samples. In plates with two or more drops, the macromolecule:cocktail ratio can be set at various values. Studies have also been performed to compare crystallization outcomes between vapor diffusion and microbatch-under-oil (Chayen, 1998 ▸). Selecting the appropriate crystallization technique is sample-dependent and sometimes involves trial and error.

**Figure 5:**
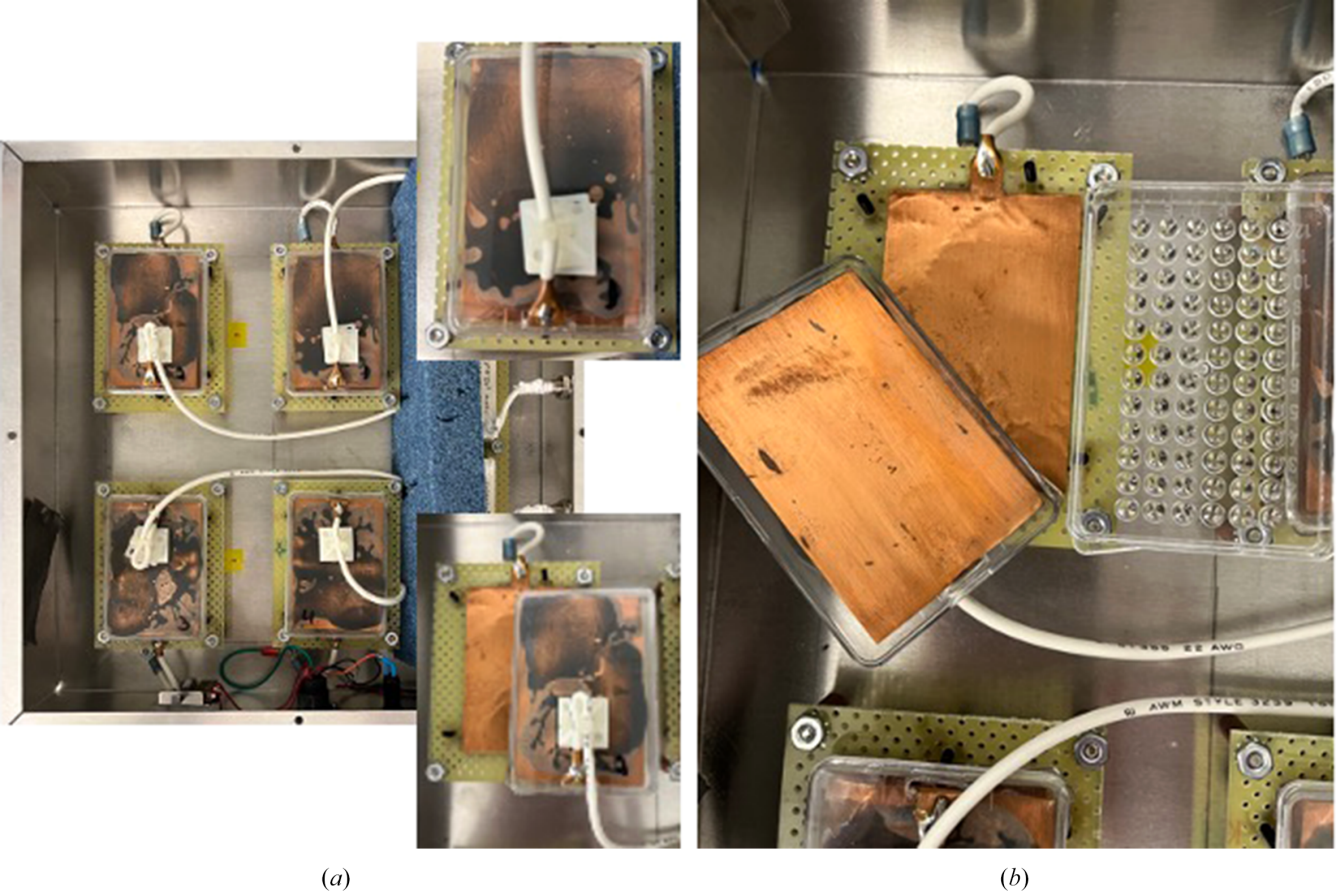
Microbatch crystallization in an electric field. (*a*) Overview of the electric field device used with microbatch plates showing the four ‘ports’ available for DC voltage application: top right, port in closed configuration; bottom right, port in open configuration. (*b*) Open ‘port’ showing the leads and microbatch plate ready for insertion between leads.

There are additional methods of crystallization, including batch (Chayen *et al.*, 1990 ▸; D’Arcy *et al.*, 1996 ▸), dialysis (Zeppezauer, 1971 ▸; Thomas *et al.*, 1989 ▸) and other specialized methods. Recently even electromagnetic fields (Frontana-Uribe & Moreno, 2008 ▸) have been effectively used to control and manipulate the crystallization process. Electric fields affect the force field between protein molecules, influencing the nucleation process and the quality of the resulting crystals. Following the initial paper by Taleb *et al.* (1999 ▸), several studies have addressed various strategies to control the kinetics and transport phenomena in the crystallization process. The method takes advantage of the batch crystallization method, positioning the electrodes in contact with the protein solution (for a recent review, see Alexander & Radacsi, 2019 ▸). Despite the observed nucleation reduction and crystal quality improvement, this method has some challenges: the batch method requires significant availability of pure protein, and a dedicated device that combines the batch method with electric fields is lacking. Rubin and coworkers combined the microbatch method (Chayen *et al.*, 1992 ▸) with a device that permitted the discretionary application of DC electric fields. The device used by these authors is depicted in Fig. 5 ▸; five microbatch crystallization plates (Hampton Research) are prepared simultaneously under exactly the same conditions. Four plates, one each, are inserted between the two electrodes available on each port and submitted to 1, 2.3, 4.1 and 6 kV. The fifth plate is used as control and is not exposed to an electric field (Rubin *et al.*, 2017 ▸). Although high-quality large (>100 µm) to small (<5 µm) crystals were obtained, the use of electric or even magnetic fields for protein crystallization is challenging and has not been widely used or implemented. There are two major challenges. One is the perception that large amounts of protein are required; with the availability of current crystallization robots the quantities needed are greatly reduced. Secondly, and probably the most important, is the lack of standard devices; a large variety of ‘homemade’ devices with diverse geometry can be encountered in the literature. These factors are further compounded with lack of proper simulations, and therefore our understanding of the molecular interactions and the electric field are limited.

Integral membrane proteins have long been considered highly challenging to crystallize, as a result of significant tracts of hydrophobic membrane-interacting residues which make these proteins challenging to stabilize in a purely aqueous solution. Efforts to stabilize integral membrane proteins for crystallization by introducing exogenous lipids including bicelles and nanodiscs have made this protein class more tractable for crystallization (Faham & Bowie, 2002 ▸; Nikolaev *et al.*, 2017 ▸; Shelby *et al.*, 2020 ▸). Additionally, lipidic cubic phase crystallization, involving a mixture of lipids and water that generates a liquid-crystal array studded with aqueous channels, has also been implemented in the last 30 years and is now facilitated by liquid-handling robots (Landau & Rosenbusch, 1996 ▸; Caffrey, 2015 ▸; Cherezov, 2011 ▸). Other methods of crystallizing membrane proteins include the use of styrene–maleic acid copolymers, which obviate the need to use solubilizing detergents at all (Broecker *et al.*, 2017 ▸).

Seeding methods, particularly matrix microseeding (Shaw Stewart *et al.*, 2011 ▸; D’Arcy *et al.*, 2014 ▸), can be useful for improving the quality of subpar crystals or generating highly reproducible crystallizations. In this approach, parent crystals are fragmented and then included in iterative screening to reduce the entropic barrier to crystallization by providing pre-formed nucleation sites. Usefully, cross-seeding approaches can be employed wherein the parent seeds are obtained from a closely related, but not identical, molecule that has previously been crystallized (Caspy *et al.*, 2025 ▸). Several rounds of seeding can be conducted to achieve a desirable outcome and seeds can be stored to repeat crystal-growth experiments in the future.

## 5. Future outlook for crystal-based structural biology

The growth of competing and complementary experimental structural techniques, including both single-particle cryo-electron microscopy (cryo-EM) and the advent of highly accurate protein structure prediction, have altered the scope of biomolecular crystallography projects. Because of the bottleneck in finding appropriate crystallization conditions, structural solutions by crystallographic methods can be somewhat elusive for uncharacterized systems, making both structure prediction and alternative methods appealing. However, crystal-based structural methods have and will continue to play a key role in structural solution and analysis because of a few major strengths of the technique.

In drug-discovery efforts, ligand-bound structures are desirable to understand the mechanism by which a ligand binds to influence the function of a biomolecule. Understanding ligand-bound structures can also lend support to rational drug-design campaigns (Zheng *et al.*, 2014 ▸; Mazzorana *et al.*, 2020 ▸). Another key tool for rational drug-design efforts are fragment-based screens, in which crystals of a target protein are grown or soaked in the presence of small-molecule moieties that represent pieces of a larger final scaffold (Murray & Rees, 2009 ▸; Erlanson *et al.*, 2016 ▸). X-ray crystallography is well suited to capture the low-affinity binding events with which these fragments bind to biomolecules that may go undetected using common biophysical screening assays, and inform directly on the mechanism of interaction of the fragment, as well as its occupancy in the structure. Advances in robotics, liquid handling and the throughput of synchrotron sources make fragment-based screening a tenable option that is especially well suited for hard-to-target biomolecules. Although it is possible to determine ligand-bound structures with single-particle cryo-EM, it is not high throughput and often lacks the appropriate resolution to unambiguously define a binding event. Additionally, the averaging of particles necessary to solve a structure by single-particle cryo-EM makes it unlikely that partial occupancy ligands will be identified as routinely as with crystal-based methods. These limitations in cryo-EM are beginning to be addressed with new methods (Muenks *et al.*, 2023 ▸). The computational structure-prediction model *AlphaFold*3 is now capable of predicting the structure of biomolecules bound to ligands, promising to accelerate drug-discovery hypotheses (Abramson *et al.*, 2024 ▸). For the time being, however, experimental structures, primarily solved using X-ray crystallography, are the gold standard for defining protein–ligand interactions and testing hypothetical models generated by predictive methods.

Several emerging technologies capitalize on the use of microcrystals and nanocrystals, which were previously limited in their utility for diffraction experiments (Kupitz, Grotjohann *et al.*, 2014 ▸; Shoeman *et al.*, 2023 ▸). Microfocus beams (on the order of 1 × 1.5 µm) enable the collection of data from very small crystal samples at synchrotron sources. Small-sized crystal samples have proven to be optimal for interrogation by X-ray free-electron lasers (XFELs) and in serial synchrotron experiments, where single diffraction images are collected from many individual crystals to provide a complete data set (Kern *et al.*, 2013 ▸; Kupitz, Basu *et al.*, 2014 ▸; Pearson & Mehrabi, 2020 ▸). Electron diffraction methods offer an additional emerging strategy to collect structural information from nanoscale (100–600 nm) macromolecular crystals that were previously intractable by X-ray-based methods (Nannenga & Gonen, 2019 ▸). At VMXm at Diamond Light Source, crystals are mounted on EM grids within a vacuum chamber to minimize background scatter from air at the synchrotron (Warren *et al.*, 2024 ▸). Pipelines are also being developed for crystals at room temperature, allowing structural investigation of challenging fragile crystals and time-resolved studies (Mikolajek *et al.*, 2023 ▸). Advances at XFELs using pulsed high-intensity X-ray beams allow structural determination prior to radiation damage, which is especially important for metalloproteins (Hough & Owen, 2021 ▸).

XFEL and synchrotron-based X-ray diffraction experiments are also increasingly being employed to study the dynamics of a sample *in crystallo* by perturbing the sample and observing time-resolved effects, enabling the investigation of the structure of biomolecules in action (Lin *et al.*, 2024 ▸; Smith *et al.*, 2024 ▸). Time-resolved structural experiments represent the frontier of structural biology in understanding the interplay of structure and function and promise to inform on key mechanistic details of biochemical reactions. Critical for all of these dynamic and time-resolved studies is the sample size; therefore, much time has been invested in obtaining uniformly sized microcrystals and nanocrystals. All of these cutting-edge structural methods make it possible to use nearly the entire range of potential crystal sizes to collect structural information, making crystal-based methods a more powerful and versatile tool than ever, fit to answer many of the questions posed by modern structural biology.

## 6. Concluding thoughts

Sample preparation is a critical part of generating crystals for structural biology. It can often take some trial and error to find the correct sample construct, the optimal crystallization cocktail and the best method of crystallization. When confronting difficulties in crystallizing biomolecules, we recommend asking advice from experienced groups or facility staff, especially for new crystallographers. Facilities are also available to provide access to both expertise and to a wide range of crystallization reagents, plate types and robotics (Stegmann *et al.*, 2023 ▸; Budziszewski *et al.*, 2023 ▸; Sandy *et al.*, 2024 ▸).

## Figures and Tables

**Table 1:** Solution half-lives of common biochemical reducing agents

Chemical reductant	Solution half-life (h)
Dithiothreitol (DTT)	40 h (pH 6.5), 1.5 h (pH 8.5)
β-Mercaptoethanol (BME)	100 h (pH 6.5), 4.0 h (pH 8.5)
Tris(2-carboxyethyl)phosphine hydrochloride (TCEP)	>500 h† (pH 1.5–11.1)

† In nonphosphate buffers.
